# β-Lactam vs Non–β-Lactam Prophylaxis in Elective Colorectal Surgery

**DOI:** 10.1001/jamanetworkopen.2026.6708

**Published:** 2026-04-13

**Authors:** Curtis D. Collins, Eric Hartsfield, Robert K. Cleary, Michael P. Veve, Kara K. Brockhaus

**Affiliations:** 1 Department of Pharmacy Services, Trinity Health Ann Arbor, Michigan; 2 Department of Colon and Rectal Surgery, Trinity Health Ann Arbor, Michigan; 3 Department of Pharmacy, Henry Ford Hospital, Detroit, Michigan; 4 Department of Pharmacy Practice, Eugene Applebaum College of Pharmacy and Health Sciences, Wayne State University, Detroit, Michigan

## Abstract

**Importance:**

Previous studies found reduced surgical site infections (SSIs) with β-lactam surgical infection prophylaxis compared with non–β-lactam alternatives; however, variations in timing and dosing have been reported. Whether guideline-concordant preoperative antibiotic dosing or timing are associated with reported SSI differences between regimens is unclear.

**Objective:**

To evaluate the incidence of SSIs between patients receiving β-lactam and non–β-lactam alternative prophylaxis, while controlling guideline-concordant dosing and timing.

**Design, Setting, and Participants:**

This multicenter retrospective cohort study used data from a validated regional data registry. Adult patients (>18 years) receiving β-lactam and non–β-lactam alternative prophylaxis for elective colorectal surgery between July 2012 and June 2021 were included. Analyses were performed at multiple intervals between March 2022 and January 2026.

**Exposures:**

Surgical prophylaxis regimens with β-lactam or non–β-lactam alternative prophylaxis. Receipt of guideline-concordant prophylaxis dose and timing were variables of interest, defined using international guideline recommendations.

**Main Outcomes and Measures:**

The primary outcome was 30-day SSI. Modified Poisson regression with robust (sandwich) variance estimators was used to estimate adjusted risk ratios (ARRs) for SSI comparing β-lactam with non–β-lactam alternative prophylaxis, adjusting for guideline-concordant dosing, timing, and other covariates; 1:1 propensity score matching was performed as a sensitivity analysis.

**Results:**

Among 20 140 procedures (female, 10 423 [51.8%]; male, 9717 [48.2%]; mean [SD] age, 62.0 [13.8] years), 18 098 (89.9%) received β-lactam prophylaxis and 2042 (10.1%) received non–β-lactam alternative prophylaxis. Baseline characteristics differed between groups. Patients in the β-lactam cohort more commonly received guideline-concordant antibiotic dosing (15 124 [83.6%] vs 1084 [53.1%]; *P* < .001), guideline-concordant timing (16 946 [93.6%] vs 1707 [83.6%]; *P* < .001), or combined guideline-concordant dose and timing (14 216 [78.6%] vs 751 [36.8%]; *P* < .001), and had fewer SSIs (1114 [6.2%] vs 172 [8.4%]; *P* < .001). After adjusting for confounders, β-lactam prophylaxis was associated with a lower risk of SSIs (ARR, 0.74; 95% CI, 0.63-0.87; *P* < .001). Neither guideline-concordant dosing (ARR, 1.04; 95% CI, 0.91-1.20; *P* = .54) nor timing (ARR, 1.13; 95% CI, 0.92-1.38; *P* = .25) were associated with SSI risk.

**Conclusions and Relevance:**

In this cohort study of elective colorectal surgery procedures, β-lactam surgical prophylaxis was associated with significantly lower SSI risk compared with non–β-lactam alternatives. Differences in guideline-concordant dosing and timing between cohorts were not associated with SSI risk in adjusted analyses.

## Key Points

**Question:**

Are surgical prophylaxis regimens containing β-lactam antibiotics associated with lower surgical site infections (SSIs) compared with non–β-lactam alternative regimens in patients undergoing colorectal operations when considering antibiotic timing and dose optimization?

**Findings:**

In this cohort study including 20 140 procedures in adult patients receiving β-lactam and non–β-lactam alternative prophylaxis for elective colorectal surgery, the use of β-lactam prophylaxis regimens was associated with increased guideline-concordant prophylaxis dosing and timing and reduced SSIs.

**Meaning:**

This study suggests that, regardless of differences in guideline-concordant dosing and timing, β-lactam surgical prophylaxis is associated with lower risk of SSIs compared with those who receive non–β-lactam alternative regimens.

## Teaser

This cohort study reports on the occurrence of surgical site infections with surgical prophylaxis regimens that use β-lactam antibiotics compared with those using non–β-lactam alternative regimens in patients undergoing colorectal operations.

## Introduction

Surgical site infections (SSIs) are a substantial health care burden, associated with increased patient morbidity and up to $10 billion in annual health care costs.^1,2,3,4,5,6,7,8^ β-Lactam antibiotics are preferred for surgical infection prophylaxis over non–β-lactam alternatives due to their appropriate spectrum of activity and potency, favorable pharmacokinetics and half-life for procedural redosing, relative safety, and low cost.^4^ Previous studies have identified that patients with a documented penicillin or β-lactam allergy are frequently prescribed a non–β-lactam alternative for surgical prophylaxis.^9,10,11,12^ This is often due to concern for cross-reactivity between β-lactams, despite the evidence that the risk of allergic reactions between antibiotics with dissimilar R1 side chains is negligible and that the use of non–β-lactam prophylaxis has been associated with higher SSI rates.^13,14,15,16,17,18,19,20,21,22,23,24^ The association of decreased SSIs observed with β-lactam prophylaxis has also been reported in colorectal surgical procedures, which are among the procedures with the highest SSI risk.^4,16,22^

The underlying reasons for the reported advantage of β-lactam prophylaxis in reducing SSIs are not well understood. Theories include their reliable coverage of common surgical pathogens, favorable pharmacokinetic properties, and the added advantages of dosing simplicity and prescriber familiarity.^4,16^ Antibiotic timing and dosing are key modifiable factors incorporated into guidelines and quality measures to ensure enhanced drug and tissue exposure prior to incision.^1,2,3,4,5,6,24,25,26,27,28,29,30,31,32^ Previous studies observing differences in SSIs between β-lactam and non–β-lactam alternatives have not simultaneously controlled for both technical aspects of surgical prophylaxis.^9,10,16,17,18,19,20,21^ Therefore, it is plausible that observed SSI differences between regimens have been confounded by variations in dosing and adherence, particularly in agents with lower familiarity and more complex dosing, rather than an inherent lack of efficacy. The purpose of this study was to investigate whether β-lactam vs non–β-lactam alternative preoperative surgical infection prophylaxis is associated with SSIs after controlling for guideline-concordant dosing and timing.

## Methods

### Design

This multicenter retrospective cohort study of patients undergoing elective colorectal surgery between July 2012 and June 2021 was completed using data collected from the Michigan Surgical Quality Collaborative (MSQC) database.^16,17,30,33,34^ The study cohorts included patients who underwent an elective colorectal surgery who received β-lactam or non–β-lactam alternatives as surgical infection prophylaxis.

The primary objective of this study was to determine the proportion of patients who develop 30-day SSI between β-lactam and non–β-lactam alternatives for patients undergoing elective colorectal surgery, while controlling for guideline-concordant preoperative prophylaxis dosing and timing. This study followed the Strengthening the Reporting of Observational Studies in Epidemiology (STROBE) reporting guideline and received approval with waiver of Health Insurance Portability and Accountability Act authorization from the Trinity Health Ann Arbor Institutional Review Board. Informed consent was waived as secondary research of existing data. Analyses were performed at multiple intervals between March 2022 and January 2026.

### Population

This study was completed using data available within the MSQC, a validated database that contains deidentified patient-level data from 73 hospitals in Michigan.^33,34^ The MSQC includes community and academic hospitals in urban and rural settings and uses trained abstractors with standardized definitions and routine audit processes to promote data reliability.

Patients were included if they were 18 years or older, had elective colorectal surgery during the study period, and received surgical prophylaxis. Patients were excluded if their surgery was urgent or emergent, had a clean (class I) or dirty/infected (class IV) wound classification, underwent concomitant small-bowel procedures, had missing data for any study variable (including 30-day outcome status, antibiotic selection, dosing, timing, mechanical and oral antibiotic bowel preparation [MOABP] receipt, or height/weight), if prophylaxis timing was more than 3.5 hours before incision or more than 1 hour after incision, or if body mass index (BMI [calculated as weight in kilograms divided by height in meters squared]) was 70 or greater or less than 15 (eFigure in Supplement 1).^30,34^ Patients who received antibiotics outside of the Surgical Care Improvement Project or American Society of Health-System Pharmacists guidelines were excluded.^1,4^ Patients who received clindamycin plus aztreonam were excluded to avoid hybrid β-lactam plus non–β-lactam regimens, and procedures with documented surgery duration of less than 1 hour or more than 8 hours were excluded to account for possible surgical duration data entry errors.

### Variables and Definitions

With the exception of guideline-concordant antibiotic dosing and timing, procedural coding, demographic and clinical characteristics, and outcome definitions (ie, 30-day SSIs, and *Clostridioides difficile* infection [CDI]) were defined according to previously reported MSQC standards.^30,34^ The β-lactam prophylaxis cohort was defined as patients receiving guideline-concordant prophylaxis regimens including cefazolin plus metronidazole, cefoxitin, cefotetan, ampicillin-sulbactam, ceftriaxone plus metronidazole, and ertapenem.^4^ The non–β-lactam alternative prophylaxis cohort consisted of patients receiving guideline-concordant clindamycin plus an aminoglycoside or a fluoroquinolone and metronidazole plus an aminoglycoside or a fluoroquinolone.^4^ Patient demographic characteristics included age, sex, race, BMI, weight, height, diabetes status, smoking status, corticosteroid use, wound classification, American Society of Anesthesiologists (ASA) classification, procedural groupings and approach, surgery duration, and receipt of MOABP.^30,34^

Race was abstracted in the MSQC registry from the medical record by trained clinical data abstractors and may reflect patient self-identification or assignment per local hospital policy.^30,34^ Race categories were recorded as White and other, with other including American Indian or Alaska Native, Asian, Black or African American, Native Hawaiian or Other Pacific Islander, and unknown race. Race is presented as binary classification from predetermined definitions from the MSQC collaborative, and no further breakdown is available for classifications within the other description. Race was assessed to describe the study population and included as a covariate given that race-associated differences in perioperative care and outcomes have been reported.^35,36^

Patient outcomes, including 30-day SSI and CDI, were defined according to MSQC standards.^30,34^ Guideline-concordant antibiotic dosing and timing were defined according to international guidelines.^1,4,31^ Antibiotic-specific doses were determined to meet guideline-concordant dosing when the cumulative doses received between 3.5 hours before incision and up to 1 hour after incision met guideline recommendations.^4^ Secondary analyses investigated variations in prophylaxis dosing, including metronidazole doses up to 1 g, levofloxacin doses up to 750 mg, 3-g cefazolin dosing if the patient weighed less than 120 kg, and expanded gentamicin and tobramycin weight-based dosing (ie, 1.8 mg/kg to 7 mg/kg) (eTable 1 in Supplement 1). Guideline-concordant timing was defined as prophylaxis initiated within 60 minutes before incision or 120 minutes for fluoroquinolones. If combination regimens were used, both agents were required to meet previously described dosing and timing definitions. Prophylaxis dosing received postincision was noted.

Secondary analyses investigated the incidence of SSIs in subgroups of patients receiving MOABP, patients receiving MOABP with guideline-concordant dosing and timing, patients receiving individual prophylaxis regimens, and patients receiving combination vs single agent prophylaxis. A propensity score sensitivity analysis was performed to match groups on variables associated with 30-day SSI.

### Statistical Analysis

Patient characteristics and outcomes were summarized using means (SDs) or medians (IQRs) for continuous variables and counts (percentages) for categorical variables. Modified Poisson regression models with a log link and robust (sandwich) variance estimators were used to estimate adjusted relative risks (ARRs) of 30-day SSI. Covariates included prophylaxis (β-lactam vs non–β-lactam alternative), guideline-concordant dosing or timing, age, sex, race, BMI, diabetes status, smoking status, corticosteroid use, wound classification, ASA scoring, procedural grouping, surgical approach, surgery duration, and receipt of MOABP. Covariate coefficients are presented to describe adjusted associations within the specified model and not interpreted as causal effects for individual covariates. Additional modified Poisson regression modeling evaluated relevant patient subgroups, including variations in dosing, patients who received MOABP, and prophylaxis regimen.

Propensity score matching was performed 1:1 without replacement using a caliper (maximum allowable absolute difference) of 0.05 on the propensity score (probability) scale. Propensity scores were estimated using logistic regression including the baseline covariates (age, sex, race, BMI, diabetes, smoking, corticosteroid use, wound class, ASA class, procedure group, surgical approach, surgical duration, and MOABP) excluding guideline-concordant dosing and timing. Covariate balance in the matched cohort was assessed using absolute standardized mean differences. Matched cohorts were reevaluated using modified Poisson regression with log link and robust (sandwich) variance estimators. Data were analyzed using IBM SPSS Statistics, version 26.0 (IBM Corporation), and statistical significance was defined as 2-sided *P* < .05 for all calculations.

## Results

Among 35 138 eligible cases, 20 140 procedures met the inclusion criteria after exclusions for antibiotic selection and incomplete data (eFigure in Supplement 1). Overall, 18 098 (89.9%) received β-lactam prophylaxis and 2042 (10.1%) received non–β-lactam prophylaxis. Baseline characteristics differed between groups, with the β-lactam cohort tending to be younger (mean [SD], 61.8 [13.7] vs 63.3 [13.5] years; *P* < .001), more often male (8992 [49.7%] vs 725 [35.5%]; *P* < .001) and current smokers (3886 [21.4%] vs 397 [19.4%]; *P* = .04), and less often White (14 431 [79.7%] vs 1693 [82.9%]; *P* < .001), with diabetes (2946 [16.3%] vs 389 [19.0%]; *P* = .001), or receiving corticosteroids (1066 [5.9%] vs 170 [8.3%]; *P* < .001) (Table 1). Colectomies (14 386 [79.5%] vs 1585 [77.6%]) and open surgical approaches (7791 [43.0%] vs 936 [45.8%]) comprised most procedures and approaches between the β-lactam and non–β-lactam prophylaxis cohorts, respectively. There was no statistically significant difference between cohorts in receipt of MOABP (12 575 [69.5%] in the β-lactam prophylaxis cohort vs 1394 [68.3%] in the non–β-lactam prophylaxis cohort; *P* = .26). Cefazolin plus metronidazole (8412 [46.5%]) and metronidazole plus a fluoroquinolone (896 [43.9%]) were the most prescribed regimens in in the β-lactam prophylaxis and in the non–β-lactam prophylaxis cohorts, respectively.

**Table 1.** Demographic and Clinical Characteristics by Cohort^a^

Variable and label	Prophylaxis		Overall cohort (N = 20 140)	*P* value
	β-Lactam (n = 18 098)	Alternative (n = 2042)		
Age, mean (SD), y	61.8 (13.7)	63.3 (13.5)	62.0 (13.8)	<.001
Sex				
Female	9106 (50.3)	1317 (64.5)	10 423 (51.8)	<.001
Male	8992 (49.7)	725 (35.5)	9717 (48.2)	
Race and ethnicity^b^				
White	14 431 (79.7)	1693 (82.9)	16 124 (80.1)	.001
Other^c^	3667 (20.3)	349 (17.1)	4016 (19.9)	
BMI, mean (SD)	29.0 (6.5)	29.2 (6.8)	29.0 (6.5)	.30
Diabetes	2946 (16.3)	389 (19.0)	3335 (16.6)	.001
Smoker	3866 (21.4)	397 (19.4)	4263 (21.2)	.04
Corticosteroid use	1066 (5.9)	170 (8.3)	1236 (6.1)	<.001
Wound classification				
Clean/contaminated	16 003 (88.4)	1804 (88.3)	17 807 (88.4)	.92
Contaminated	2095 (11.6)	238 (11.7)	2333 (11.6)	
ASA classification				
ASA 1	234 (1.3)	18 (0.9)	252 (1.3)	<.001
ASA 2	7868 (43.5)	790 (38.7)	8658 (43.0)	
ASA 3	9348 (51.7)	1145 (56.1)	10 493 (52.1)	
ASA 4 and 5	648 (3.6)	89 (4.4)	737 (3.7)	
Procedural group				
Colectomy	14 386 (79.5)	1585 (77.6)	15 971 (79.3)	.13
Other colon procedures	2720 (15.0)	331 (16.2)	3051 (15.1)	
Proctectomy	992 (5.5)	126 (6.2)	1118 (5.6)	
Surgical approach				
Open	7791 (43.0)	936 (45.8)	8727 (43.3)	.06
Laparoscopic	6854 (37.9)	714 (35.0)	7568 (37.6)	
Robotic	3327 (18.4)	378 (18.5)	3705 (18.4)	
Other	126 (0.7)	14 (0.7)	140 (0.7)	
MOABP	12 575 (69.5)	1394 (68.3)	13 969 (69.4)	.26
Surgery duration, median (IQR)^d^	10 (7-14)	10 (7-14)	10 (7-14)	.92
Prophylaxis received				
Cefazolin and metronidazole	8412 (46.5)	NA	8412 (41.8)	NA
Cefoxitin	3749 (20.7)	NA	3749 (18.6)	NA
Ertapenem	3225 (17.8)	NA	3225 (16.0)	NA
Ceftriaxone and metronidazole	914 (5.1)	NA	914 (4.5)	NA
Ampicillin and sulbactam	985 (5.4)	NA	985 (4.9)	NA
Cefotetan	813 (4.5)	NA	813 (4.0)	NA
Metronidazole and fluoroquinolone	NA	896 (43.9)	896 (4.4)	NA
Clindamycin and aminoglycoside	NA	787 (38.5)	787 (3.9)	NA
Metronidazole and aminoglycoside	NA	237 (11.6)	237 (1.2)	NA
Clindamycin and fluoroquinolone	NA	122 (6.0)	122 (0.6)	NA
Guideline-concordant dosing and timing				
Guideline-concordant dosing	15 124 (83.6)	1084 (53.1)	16 208 (80.5)	<.001
Guideline-concordant dosing (expanded aminoglycoside dosing)	15 124 (83.6)	1335 (65.4)	16 459 (81.7)	<.001
Guideline-concordant dosing (expanded aminoglycoside, cefazolin, fluoroquinolone, and metronidazole dosing)	15 680 (86.6)	1555 (76.2)	17 235 (85.6)	<.001
Guideline-concordant timing	16 946 (93.6)	1707 (83.6)	18 653 (92.6)	<.001
Guideline-concordant dosing and timing	14 216 (78.6)	751 (36.8)	14 967 (74.3)	<.001
Guideline-concordant dosing and timing (expanded aminoglycoside dosing)	14 216 (78.6)	1120 (54.8)	15 336 (76.1)	<.001
Guideline-concordant dosing and timing (expanded aminoglycoside, cefazolin, fluoroquinolone, and metronidazole dosing)	14 709 (81.3)	1297 (63.5)	16 006 (79.5)	<.001
At least 1 preoperative antibiotic given postincision	494 (2.7)	198 (9.7)	692 (3.4)	<.001
All preoperative prophylaxis postincision	179 (0.9)	11 (0.5)	190 (0.9)	.046

Abbreviations: ASA, American Society of Anesthesiologists; BMI, body mass index (calculated as weight in kilograms divided by height in meters squared); MOABP, mechanical and oral antibiotic bowel preparation; NA, not applicable.

^a^ Data are presented as No. (%) unless otherwise specified.

^b^ Race was recorded in the Michigan Surgical Quality Collaborative (MSQC) registry from the medical record by trained clinical data abstractors and may reflect patient self-identification or assignment per local hospital policy. Race is presented as binary classification from predetermined definitions from the MSQC collaborative.

^c^ Other includes American Indian or Alaska Native, Asian, Black or African American, Native Hawaiian or Other Pacific Islander, and unknown race. No numeric breakdown for this population is available.

^d^ Surgical time divided by and rounded to the nearest 15 minutes.

Patients in the β-lactam prophylaxis cohort compared with those in the non–β-lactam prophylaxis cohort more commonly received guideline-concordant antibiotic dosing (15 124 [83.6%] vs 1084 [53.1%]; *P* < .001) and timing (16 946 [93.6%] vs 1707 [83.6%]; *P* < .001). When considering guideline-concordant antibiotic timing and dosing as a composite variable, patients receiving β-lactam prophylaxis were significantly more likely to receive both guideline-concordant dosing and timing (14 216 [78.6%] vs 751 [36.8%]; *P* < .001) and less likely to have received at least 1 preoperative antibiotic postincision (494 [2.7%] vs 198 [9.7%]; *P* < .001).

Guideline concordance varied by antibiotic selection (eTable 2 in Supplement 1). Of all prophylaxis regimens used in either cohort, clindamycin and aminoglycoside regimens demonstrated the lowest concordance. Of 787 clindamycin and aminoglycoside regimens received, 187 (23.8%) received guideline-concordant dosing and 660 (83.9%) received concordant timing, yielding 70 (8.9%) with both guideline-concordant dosing and timing. This improved to 312 (39.6%) using expanded dosing definitions.

The incidence of SSIs was significantly lower in the β-lactam cohort compared with the non–β-lactam prophylaxis cohort (1114 [6.2%] vs 172 [8.4%]; *P* < .001) (Table 2). This remained consistent when limited to only patients who received MOABP (634 [5.0%] vs 92 [6.6%]; *P* = .01) and those who received MOABP with guideline-concordant dosing and timing (502 [5.0%] vs 44 [8.1%]; *P* = .001). There was no statistically significant difference between cohorts in the development of CDI (176 [1.0%] vs 24 [1.2%]; *P* = .38).

**Table 2.** Outcomes by Cohort^a^

Surgical site infection	Prophylaxis		Overall cohort (N = 20 140)	*P* value
	β-Lactam (n = 18 098)	Alternative (n = 2042)		
Overall study population	1114 (6.2)	172 (8.4)	1286 (6.4)	<.001
Deep surgical site infection	146 (0.8)	26 (1.3)	172 (0.9)	.03
Organ space surgical site infection	495 (2.7)	66 (3.2)	561 (2.8)	.20
Superficial surgical site infection	493 (2.7)	83 (4.1)	576 (2.9)	.001
Surgical site infection with receipt of MOABP^b^	634 (5.0)	92 (6.6)	726 (5.2)	.01
Surgical site infection with receipt of MOABP and guideline-concordant dosing and timing^c^	502 (5.0)	44 (8.1)	546 (5.2)	.001
*Clostridioides difficile* infection	176 (1.0)	24 (1.2)	200 (1.0)	.38

Abbreviation: MOABP, mechanical and oral antibiotic bowel preparation.

^a^ Data are presented as No. (%).

^b^ Analysis performed on 13 969 procedures (12 575 β-lactam prophylaxis, 1394 alternative prophylaxis) with receipt of MOABP.

^c^ Analysis performed on 10 521 procedures (9981 β-lactam prophylaxis, 540 alternative prophylaxis) with receipt of MOABP and with receipt of guideline-concordant dosing and timing.

Following adjustment for covariates, results of the modified Poisson regression model showed that receipt of β-lactam prophylaxis was associated with a lower risk of SSIs (ARR, 0.74; 95% CI, 0.63-0.87; *P* < .001; Table 3). Guideline-concordant dosing (ARR, 1.04; 95% CI, 0.91-1.20; *P* = .54) and guideline-concordant timing (ARR, 1.13; 95% CI, 0.92-1.38; *P* = .25) were not associated with SSI risk. Table 3 presents ARRs for all covariates included in the model, and coefficients for covariates are not interpreted as causal effects.

**Table 3.** Association With Surgical Site Infection (Modified Poisson Regression)

Variable	Adjusted risk ratio (95% CI)	*P* value
β-Lactam prophylaxis	0.74 (0.63-0.87)	<.001
Guideline-concordant dosing	1.04 (0.91-1.20)	.54
Guideline-concordant timing	1.13 (0.92-1.38)	.25
MOABP	0.59 (0.53-0.66)	<.001
Age	0.99 (0.99-1.00)	.001
Sex		
Male	1 [Reference]	.78
Female	0.99 (0.89-1.09)	
Race and ethnicity^a^		
White	1.05 (0.92-1.19)	.49
Other^b^	1 [Reference]	
BMI	1.02 (1.02-1.03)	<.001
Diabetes	1.06 (0.92-1.22)	.44
Smoker	1.16 (1.03-1.32)	.02
Corticosteroid use	1.28 (1.06-1.54)	.01
Wound classification^c^		
Contaminated	1.30 (1.13-1.50)	<.001
ASA classification^d^		
ASA 3 and 4	1.22 (1.09-1.37)	.001
Procedural group^e^		
Other colon procedures	1.19 (1.04-1.36)	.01
Proctectomy	1.41 (1.16-1.71)	.001
Surgical approach^f^		
Laparoscopic	0.48 (0.41-0.55)	<.001
Robotic	0.57 (0.49-0.67)	<.001
Other	0.31 (0.12-0.83)	.02
Surgery duration^g^	1.04 (1.04-1.05)	<.001

Abbreviations: ASA, American Society of Anesthesiologists; BMI, body mass index; MOABP, mechanical and oral antibiotic bowel preparation.

^a^ Race was recorded in the Michigan Surgical Quality Collaborative (MSQC) registry from the medical record by trained clinical data abstractors and may reflect patient self-identification or assignment per local hospital policy. Race is presented as binary classification from predetermined definitions from the MSQC collaborative.

^b^ Other includes American Indian or Alaska Native, Asian, Black or African American, Native Hawaiian or Other Pacific Islander, and unknown race. No numeric breakdown for this population is available.

^c^ The baseline value for wound classification is number of clean/contaminated procedures.

^d^ The baseline value for ASA classification is an ASA score of 1 or 2.

^e^ The baseline value for procedural group is number of colectomy procedures.

^f^ The baseline value for surgical approach is number of open procedures.

^g^ Surgical time divided by 15 minutes.

Secondary analyses investigating variations in dosing showed no dosing variations changed the results; β-lactam prophylaxis was associated with lower SSI risk and guideline-concordant dosing and timing remained nonsignificant. These included expanded aminoglycoside dosing (ie, 1.8 to 7 mg/kg; ARR, 1.06; 95% CI, 0.92-1.21; *P* = .42) and expanded aminoglycoside, cefazolin, fluoroquinolone, and metronidazole dosing analyses (ARR, 1.03; 95% CI, 0.88-1.19; *P* = .74).

In analyses limited to patients who received MOABP, β-lactam prophylaxis was associated with lower SSI risk (ARR, 0.79; 95% CI, 0.63-0.98; *P* = .03), while neither receipt of guideline-concordant dosing (ARR, 0.98; 95% CI, 0.81-1.18; *P* = .82) nor timing (ARR, 1.10; 95% CI, 0.84-1.44; *P* = .49) were associated with SSIs (eTables 3-5 in Supplement 1). No analysis of alternative dosing changed the results.

Ertapenem (ARR, 1.20; 95% CI, 1.02-1.40; *P* = .03), cefoxitin (ARR, 1.16; 95% CI, 1.00-1.34; *P* = .47), clindamycin and aminoglycoside (ARR, 1.64; 95% CI, 1.29-2.08; *P* < .001), metronidazole and aminoglycoside (ARR, 1.61; 95% CI, 1.07-2.41; *P* = .02), and clindamycin and fluoroquinolones (ARR, 2.06; 95% CI, 1.29-3.30; *P* = .002) were associated with increased SSIs compared with cefazolin and metronidazole (eTable 6 in Supplement 1). There were no SSI differences in other prophylaxis regimens vs cefazolin and metronidazole. These differences were also found when expanded dosing regimens were analyzed. There were no significant differences between regimens and cefazolin and metronidazole in the subset of patients who received MOABP.

Full study cohort analyses demonstrated that patients who received combination regimens were less likely than those who received single agent prophylaxis to receive guideline-concordant dosing (8467 [74.5%] vs 7741 [88.2%]; *P* < .001), guideline-concordant timing (10 171 [89.5%] vs 8482 [96.7%]; *P* < .001), and combined dosing and timing (7479 [65.8%] vs 7488 [85.4%]; *P* < .001). Despite these differences, SSIs were significantly lower with combination prophylaxis (667 [5.9%] vs 619 [7.1%]; *P* = .001).

In a propensity score–matched sensitivity cohort (2042 pairs; 4084 procedures), SSI incidence was lower with β-lactam prophylaxis (123 [6.0%] vs 172 [8.4%]; *P* = .003) (eTables 7 and 8 in Supplement 1). In modified Poisson regression, β-lactam prophylaxis was associated with lower SSI risk (ARR, 0.74; 95% CI, 0.59-0.94; *P* = .01), while guideline-concordant dosing (ARR, 1.09; 95% CI, 0.86-1.38; *P* = .48) and guideline-concordant timing (ARR, 1.10; 95% CI, 0.79-1.53; *P* = .59) were not associated with SSI risk (eTable 9 in Supplement 1).

## Discussion

In this statewide, multicenter cohort of 20 140 elective colorectal procedures, β-lactam prophylaxis was associated with fewer SSIs compared with non–β-lactam alternatives. Although recipients of β-lactam prophylaxis were more likely to receive prophylaxis consistent with guideline-concordant dosing and timing, these factors were not independently associated with an increased risk of SSI after adjustment for covariates. Rather, receipt of β-lactam prophylaxis was independently associated with a lower risk of SSI. These findings highlight the potential opportunity for antimicrobial stewardship interventions to optimize perioperative surgical prophylaxis. The 2022 Allergy Practice Parameters from the American Academy of Allergy, Asthma, and Immunology and the American College of Allergy, Asthma, and Immunology outline that β-lactam regimens can be safely selected in all but rare exceptions.^14^ Knowing this, beyond the need for improving low adherence to dosing and timing standards, strategies such as structured allergy assessment, side-chain–based β-lactam selection, combined with active pharmacist involvement, can safely expand first-line β-lactam use, align care with evidence-based guidelines, and enhance patient outcomes.^13^

The present study findings build on the growing body of literature reporting an efficacy advantage of β-lactam prophylaxis in comparison with alternative therapies.^9,10,16,17,18,19,20,21,24^ Additionally, this study is one of few to assess SSI differences while controlling for both guideline-concordant dosing and timing. Blumenthal and colleagues^9^ reported 50% higher odds of SSIs attributable to the receipt of second-line perioperative antibiotics (adjusted odds ratio [OR], 1.51; 95% CI, 1.02-2.22) among 1250 patients who underwent colorectal procedures, 10.1% of whom developed SSIs. Procedure-specific prophylaxis and dosing information was not detailed; overall, β-lactam regimens were more often initiated within 2 hours prior to incision (98.2% vs 96.0%; *P* < .001), but adherence within 1 hour was not reported. Similarly, Lam and colleagues^10^ found increased SSIs among patients with a self-reported β-lactam allergy in noncolorectal procedures, which the authors concluded was attributable to alternative prophylaxis. Recently, a large, nationwide cohort study from Switzerland found that non–β-lactam prophylaxis was independently associated with 1.8-fold greater odds of SSIs across all procedure types, including colorectal procedures.^24^ Timing breakdowns in 25-minute increments within 120 minutes prior to incision were listed while antibiotic dosing was not reported. Previous MSQC-based studies evaluating antibiotic selection and SSIs support the findings of the present study. Hendren et al^17^ observed a lower odds of SSIs with cefazolin plus metronidazole, ertapenem, or ciprofloxacin plus metronidazole when compared with alternative antibiotics (adjusted OR, 0.67; 95% CI, 0.47-0.97), while timing adherence of more than 95% did not influence outcomes. Additionally, Kuriakose et al^16^ reported significantly fewer SSIs when β-lactams were used compared with non–β-lactam antibiotics after colectomy (OR, 1.65; 95% CI, 1.20-2.26). Notably, none of these studies adjusted for dosing or timing, underscoring the gap covered in the present analysis.^16^

Most guidelines recommend antibiotic administration within 60 minutes of incision, except for agents requiring prolonged infusion; however, World Health Organization guidelines and systematic reviews suggest that evidence does not support narrowing the window to less than 2 hours before incision.^2,3,4,5,25,26,27,37^ Higher dosing adherence observed with β-lactam regimens may reflect clinician familiarity and clearer guideline recommendations compared with aminoglycoside-based alternatives, which have variable dosing strategies. Lower gentamicin concentrations and inconsistent dosing (eg, 1.5 mg/kg) during prolonged colorectal procedures (>3.5 hours) have been associated with increases in SSI risk.^4,38,39^ Despite these differences, neither standard nor expanded dosing definitions (ie, 1.8-7 mg/kg) altered SSI outcomes in our analysis.

### Limitations

Several limitations require acknowledgment. This was a retrospective study that is inherently limited in ability to determine causal inferences; however, the methods used and sample size evaluated represent heterogenous surgical prophylaxis practice in Michigan. Variables of interest that may have influenced preoperative antibiotic selection, such as patient-specific culture data or documented allergies, were unavailable. To mitigate measured confounding by indication, we conducted a propensity score–matched sensitivity analysis to improve balance on observed covariates; however, propensity score methods can only account for measured variables, and residual confounding by unmeasured factors related to antibiotic selection (eg, antibiotic allergies) may persist. In addition, propensity score matching estimates associations within the matched population (ie, patients with overlapping propensity scores), which may limit generalizability to patients who could not be matched or who differ from those included in the matched cohort. Additional confounding variables, such as perioperative normothermia and perioperative glucose management, were also unavailable during the entire study time frame. Postoperative antibiotic duration was not analyzed; however, the study population only consisted of elective cases where postoperative antibiotics are often not required.^6^ Depending on operative case length and antimicrobial prophylaxis chosen, intraoperative redosing of antimicrobials may be required to maintain adequate tissue and serum concentrations.^4,40^

Enhanced recovery program adoption during the study period may have improved perioperative outcomes, including SSI reduction, but protocol variability could introduce site-level heterogeneity.^2,41,42^ In the present study, MOABP was associated with a reduction in SSIs, supporting current guideline recommendations for use in elective colorectal surgery.^2,26^ During the study period, there was a substantial oral neomycin drug shortage that may have prevented patients from receiving appropriate MOABP with subsequent impact on SSI risk. The study time frame (July 2012 and June 2021) also overlapped with the SARS-CoV-2 pandemic, and it is difficult to quantify the impact this overlap may have had on study findings. Additionally, while a post hoc analysis revealed that the large primary analysis was appropriately powered to detect SSI differences between cohorts, secondary subgroup or sensitivity analyses may have been underpowered. Finally, although MSQC data are abstracted using standardized definitions and audit processes, coding or abstraction error remains possible.

## Conclusions

In this cohort study of elective colorectal surgery procedures, β-lactam surgical prophylaxis was associated with significantly lower SSI risk compared with non–β-lactam alternatives. Recipients of β-lactam prophylaxis were more likely to receive prophylaxis consistent with guideline-concordant dosing and timing; however, these factors were not independently associated with an increased SSI risk. Results highlight the potential opportunity for continued optimization of antibiotic surgical prophylaxis.

## Notes

Supplement 1. **eFigure.** Cohort Flow Diagram **eTable 1.** Antibiotic Dosing Regimens **eTable 2.** Clinical Characteristics by Antibiotic Selection for Study Population **eTable 3.** Demographics and Clinical Characteristics by Cohort for Those with Receipt of Mechanical and Oral Antibiotic Bowel Preparation **eTable 4.** Outcomes by Cohort for Those with Receipt of Mechanical and Oral Antibiotic Bowel Preparation **eTable 5.** Association with Surgical Site Infection (Modified Poisson Regression) for Those with Receipt of Mechanical and Oral Antibiotic Bowel Preparation **eTable 6.** Poisson Regression Model Results – Prophylaxis Regimens **eTable 7.** Demographics and Clinical Characteristics in the Propensity Matched Cohort **eTable 8.** Outcomes by Cohort in the Propensity Matched Cohort **eTable 9.** Association with Surgical Site Infection (Modified Poisson Regression) in the Propensity Matched Cohort

Supplement 2.
Data Sharing Statement
