# Supplementary material for: β-Lactam vs Non–β-Lactam Prophylaxis in Elective Colorectal Surgery
Source: JAMA Netw Open. 2026 Apr 13;9(4):e266708. doi: 10.1001/jamanetworkopen.2026.6708 (PMC13077511; doi:10.1001/jamanetworkopen.2026.6708)
Supplement: Supplement 1. — eFigure. Cohort Flow Diagram eTable 1. Antibiotic Dosing Regimens eTable 2. Clinical Characteristics by Antibiotic Selection for Study Population eTable 3. Demographics and Clinical Characteristics by Cohort for Those with Receipt of Mechanical and Oral Antibiotic Bowel Preparation eTable 4. Outcomes by Cohort for Those with Receipt of Mechanical and Oral Antibiotic Bowel Preparation eTable 5. Association with Surgical Site Infection (Modified Poisson Regression) for Those with Receipt of Mechanical and Oral Antibiotic Bowel Preparation eTable 6. Poisson Regression Model Results – Prophylaxis Regimens eTable 7. Demographics and Clinical Characteristics in the Propensity Matched Cohort eTable 8. Outcomes by Cohort in the Propensity Matched Cohort eTable 9. Association with Surgical Site Infection (Modified Poisson Regression) in the Propensity Matched Cohort [file jamanetwopen-e266708-s001.pdf]

Supplemental Online Content

Collins CD, Hartsfield E, Cleary RK, Veve MP, Brockhaus KK. β-Lactam vs non-β-lactam prophylaxis in elective colorectal surgery. *JAMA Netw Open*. 2026;9(4):e266708. doi:10.1001/jamanetworkopen.2026.6708

eFigure 1. Cohort Flow Diagram
eTable 1. Antibiotic Dosing Regimens
eTable 2. Clinical Characteristics by Antibiotic Selection for Study Population
eTable 3. Demographics and Clinical Characteristics by Cohort for Those with Receipt of Mechanical and Oral Antibiotic Bowel Preparation
eTable 4. Outcomes by Cohort for Those with Receipt of Mechanical and Oral Antibiotic Bowel Preparation
eTable 5. Association with Surgical Site Infection (Modified Poisson Regression) for Those with Receipt of Mechanical and Oral Antibiotic Bowel Preparation
eTable 6. Poisson Regression Model Results – Prophylaxis Regimens
eTable 7. Demographics and Clinical Characteristics in the Propensity Matched Cohort
eTable 8. Outcomes by Cohort in the Propensity Matched Cohort
eTable 9. Association with Surgical Site Infection (Modified Poisson Regression) in the Propensity Matched Cohort

This supplemental material has been provided by the authors to give readers additional information about their work.

© 2026 Collins CD et al. *JAMA Network Open*.

eFigure 1. Cohort Flow Diagram

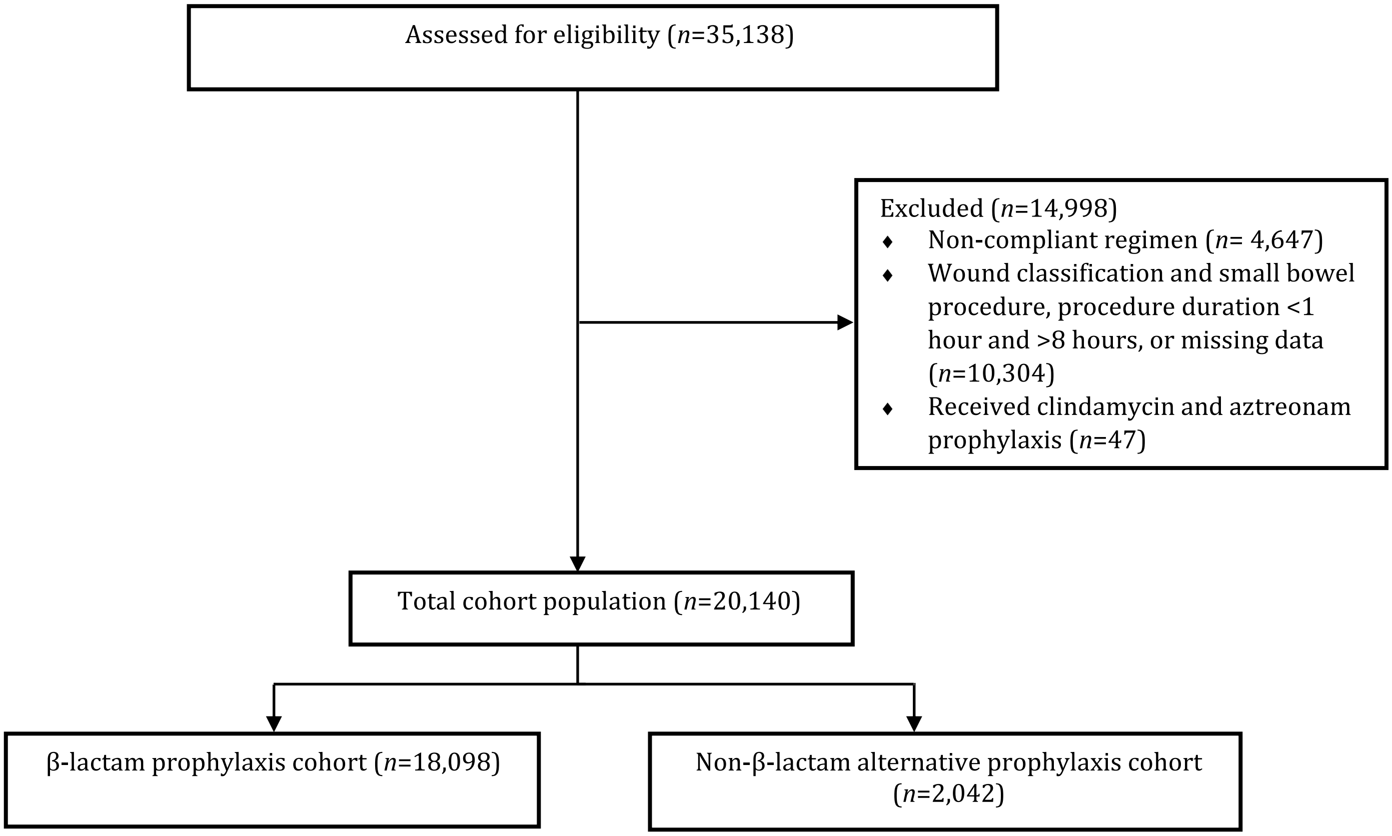

© 2026 Collins CD et al. *JAMA Network Open*.

eTable 1. Antibiotic Dosing Regimens

Antibiotic	Guideline-concordant Dosing Regimen	Additional Dosing Regimens Analyzed
Ampicillin-sulbactam	3 g	
Cefazolin	2 g (3 g in patients weighing ≥ 120kg)	3 grams in patients < 120kg
Cefoxitin	2 g	
Cefotetan	2 g	
Ceftriaxone	2 g	
Ciprofloxacin	400 mg	
Clindamycin	900 mg	
Ertapenem	1 g	
Gentamicin	5 mg/kg (dosing weight)	1.8 mg/kg - 7 mg/kg
Levofloxacin	500 mg	500 mg - 750 mg
Metronidazole	500 mg	500 mg - 1 g
Tobramycin	5 mg/kg (dosing weight)	1.8 mg/kg - 7 mg/kg

© 2026 Collins CD et al. *JAMA Network Open*.

eTable 2. Clinical Characteristics by Antibiotic Selection for Study Population

Variable[a]	Overall Cohort	Received guideline-concordant dosing	Received guideline-concordant timing	Received guideline-concordant dosing and timing	Surgical site infections
**Overall Cohort**	20,140	16,208 (80.5)	18,653 (92.6)	14,967 (74.3)	1,286 (6.4)
**β-lactam prophylaxis cohort**[b]	18,098 (89.9)	15,124 (83.6)	16,946 (93.6)	14,216 (78.6)	1,114 (6.2)
β-lactam prophylaxis cohort (expanded dosing)[b]		15,680 (86.6)		14,709 (81.3)	
Cefazolin and metronidazole	8,412 (41.8)	6,969 (82.8)	7,658 (91)	6,351 (75.5)	454 (5.4)
Cefazolin and metronidazole (expanded dosing)[c]		7,100 (84.4)		6.470 (76.9)	
Cefoxitin	3,749 (18.6)	3,290 (87.8)	3,619 (96.5)	3,183 (84.9)	279 (7.4)
Ertapenem	3,225 (16.0)	3,208 (99.5)	3,102 (96.2)	3,089 (95.8)	224 (6.9)
Ceftriaxone and metronidazole	914 (4.9)	414 (45.3)	723 (80.7)	377 (41.2)	41 (4.5)
Ceftriaxone and metronidazole (expanded metronidazole dosing)[c]		839 (93.6)		751 (83.8)	
Ampicillin and sulbactam	985 (4.9)	773 (78.5)	964 (97.9)	758 (77.0)	58 (5.9)
Cefotetan	813 (4.0)	470 (57.8)	797 (98.0)	458 (56.3)	58 (7.1)
**Alternative prophylaxis cohort**[b]	2,042 (10.1)	1,084 (53.1)	1,707 (83.6)	751 (36.8)	172 (8.4)
Alternative prophylaxis cohort (expanded dosing)[b]		1,555 (76.2)		1,297 (63.5)	
Metronidazole and fluoroquinolone	896 (4.4)	668 (74.6)	723 (80.7)	541 (60.4)	60 (6.7)
Metronidazole and fluoroquinolone (expanded dosing)[c]		881 (98.3)		712 (79.5)	
Clindamycin and aminoglycoside	787 (3.9)	187 (23.8)	660 (83.9)	70 (8.9)	76 (9.7)
Clindamycin and aminoglycoside (expanded dosing)[c]		377 (47.9)		312 (39.6)	
Metronidazole and aminoglycoside	237 (1.2)	139 (58.6)	210 (88.6)	55 (23.2)	21 (8.9)
Metronidazole and aminoglycoside (expanded dosing)[c]		200 (84.3)		182 (76.8)	
Clindamycin and fluoroquinolone	122 (0.6)	80 (73.8)	114 (93.4)	85 (69.7)	15 (12.3)

© 2026 Collins CD et al. *JAMA Network Open*.

Clindamycin and fluoroquinolone (expanded dosing)[c]	97 (79.5)	91 (74.6)

[a]Data are No. (%).
[b]Analysis performed on cohort subset.
[c]Includes metronidazole dosing up to 1 g, levofloxacin dosing up to 750 mg, cefazolin dosing up to 3 g in <120 kg, and aminoglycoside dosing 1.8-7 mg/kg.

© 2026 Collins CD et al. *JAMA Network Open*.

eTable 3. Demographics and Clinical Characteristics by Cohort for Those with Receipt of Mechanical and Oral Antibiotic Bowel Preparation

Variable and Label[a]	β-lactam prophylaxis (n=12,575)	Alternative prophylaxis (n=1,394)	Overall Cohort (n=13,969)	*P* value
Age[b]	62 (13.6)	63.1 (13.1)	62.1 (13.6)	.001
**Sex**				<.001
Female	6,276 (49.9)	895 (64.2)	7,171 (51.3)	
Male	6,299 (50.1)	499 (35.8)	6,798 (48.7)	
**Race[c]**				.005
Other	2,337 (18.6)	216 (15.5)	2,553 (18.3)	
White	10,238 (81.4)	1,178 (84.5)	11,416 (81.7)	
Body mass index, kg/m^2 [b]	29.1(6.5)	29.3 (6.9)	29.1 (6.6)	.46
Diabetes	2,094 (16.3)	261 (18.7)	2,315 (16.6)	.02
Smoker	2,621 (20.8)	264 (18.9)	2,885 (20.7)	.10
Corticosteroid use	775 (6.2)	118 (8.5)	893 (6.4)	.001
**Wound classification**				.87
Clean/Contaminated	11,041 (87.8)	1,226 (87.9)	12,267 (87.8)	
Contaminated	1,534 (12.2)	168 (12.1)	1,702 (12.2)	
**ASA classification**				.001
ASA 1	159 (1.3)	13 (0.9)	172 (1.2)	
ASA 2	5,552 (44.2)	544 (39.0)	6,096 (43.6)	
ASA 3	6,460 (51.4)	782 (56.1)	7,242 (51.8)	
ASA 4 and 5	404 (3.2)	55 (3.9)	459 (3.3)	
**Procedural group**				.48
Colectomy	10,218 (81.3)	1,121 (80.4)	11,339 (81.2)	
Other colon procedures	1,703 (13.5)	190 (13.6)	1,893 (13.6)	
Proctectomy	654 (5.2)	83 (6.0)	737 (5.3)	
**Surgical approach**				.27
Open	4,982 (39.6)	574 (41.2)	5,556 (39.8)	
Laparoscopic	4,875 (38.8)	504 (36.2)	5,379 (38.5)	
Robotic	2,654 (21.1)	310 (22.2)	2,964 (21.2)	
Other	64 (0.5)	6 (0.4)	70 (0.5)	
Surgery duration, minutes divided by 15[d, e]	10 (7, 14)	10 (8, 14)	10 (7, 14)	.70
**Prophylaxis received**				
Cefazolin and metronidazole	6,621 (52.7)		6,621 (47.4)	
Cefoxitin	2,327 (18.8)		2,327 (16.7)	
Ertapenem	1,823 (14.5)		1,823 (13.1)	
Ceftriaxone and metronidazole	850 (6.8)		850 (6.1)	
Ampicillin and sulbactam	713 (5.7)		713 (5.1)	
Cefotetan	241 (1.9)		241 (1.7)	
Metronidazole and fluoroquinolone		599 (43.0)	599 (4.3)	
Clindamycin and aminoglycoside		520 (37.3)	520 (3.7)	
Metronidazole and aminoglycoside		174 (12.5)	174 (1.2)	
Clindamycin and fluoroquinolone		101 (7.2)	101 (0.7)	
**Guideline-concordant dosing and timing**				
Guideline-concordant dosing	10,634 (84.6)	781 (56.0)	11,415 (81.7)	<.001
Guideline-concordant dosing (expanded aminoglycoside dosing)	10,634 (84.6)	962 (69.0)	11,596 (83.0)	<.001
Guideline-concordant dosing (expanded aminoglycoside, cefazolin, fluoroquinolone, and metronidazole dosing)	11,163 (88.8)	1,107 (79.4)	12,270 (87.8)	<.001

© 2026 Collins CD et al. *JAMA Network Open*.

Guideline-concordant timing	11,744 (93.4)	1,176 (84.4)	12,920 (92.5)	<.001
Guideline-concordant dosing and timing	9,981 (79.4)	540 (38.7)		<.001
Guideline-concordant dosing and timing (expanded aminoglycoside dosing)	9,981 (79.4)	814 (58.4)	10,795 (77.3)	<.001
Guideline-concordant dosing and timing (expanded aminoglycoside, cefazolin, fluoroquinolone, and metronidazole dosing)	10,449 (83.1)	934 (67.0)	11,383 (81.5)	<.001
At least one preoperative antibiotic post-incision	327 (2.6)	129 (9.3)	456 (3.3)	<.001
All preoperative prophylaxis post-incision	94 (0.7)	7 (0.5)	101 (0.7)	.31

Abbreviations: ASA, American Society of Anesthesiologists; SD, standard deviation.
[a]Data are No. (%) unless otherwise specified.
[b]Data are summarized as means (SD).
[c]Race was recorded in the MSQC registry. "Other" includes American Indian or Alaska Native, Asian, Black or African American, Native Hawaiian or Other Pacific Islander, and Unknown race.
[d]Data are summarized as median (interquartile range).
[e]Surgical time divided by and rounded to the nearest 15 minutes.

© 2026 Collins CD et al. *JAMA Network Open*.

eTable 4. Outcomes by Cohort for Those with Receipt of Mechanical and Oral Antibiotic Bowel Preparation

Variable[a]	**β-lactam prophylaxis (n=12,575)**	**Alternative prophylaxis (n=1,394)**	**Overall Cohort (n=13,969)**	***P* value**
**Surgical site infections**				
Overall population	634 (5.0)	92 (6.6)	726 (5.2)	.01
Deep surgical site infection	72 (0.6)	10 (0.7)	82 (0.6)	.50
Organ space surgical site infection	319 (2.5)	38 (2.7)	357 (2.6)	.67
Superficial surgical site infection	250 (2.0)	45 (3.2)	295 (2.1)	.002
Clostridioides difficile infection	101 (0.8)	12 (0.9)	113 (0.8)	.82

[a]Data are No. (%).

© 2026 Collins CD et al. *JAMA Network Open*.

eTable 5. Association with Surgical Site Infection (Modified Poisson Regression) for Those with Receipt of Mechanical and Oral Antibiotic Bowel Preparation

**Variable**[a]	**Adjusted risk ratio (95% CI)**	***P* value**
β-lactam prophylaxis	0.79 (0.63, 0.98)	.03
Guideline-concordant dosing	0.98 (0.81, 1.18)	.82
Guideline-concordant timing	1.10 (0.84, 1.44)	.49
Age	0.99 (0.99, 1.00)	.002
Sex, female	1.02 (0.89, 1.18)	.75
Race, white	1.08 (0.90, 1.30)	.41
Body mass index	1.03 (1.02, 1.04)	<.001
Diabetes	0.96 (0.79, 1.16)	.65
Smoker	1.22 (1.04, 1.44)	.02
Corticosteroid use	1.36 (1.07, 1.73)	.01
**Wound classification**[b]		
Contaminated	1.31 (1.09, 1.57)	.005
**ASA classification**[c]		
ASA 3 and 4	1.37 (1.17, 1.60)	<.001
**Procedural group**[d]		
Other colon procedures	1.49 (1.24, 1.79)	<.001
Proctectomy	1.53 (1.18, 1.97)	.001
**Surgical approach**[e]		
Laparoscopic	0.45 (0.37, 0.55)	<.001
Robotic	0.68 (0.56, 0.82)	<.001
Other	-	-
Surgery duration, minutes divided by 15[f]	1.05 (1.04, 1.06)	<.001

Abbreviations: CI, confidence interval; ASA, American Society of Anesthesiologists
[a]Analysis performed on 13,969 procedures with receipt of mechanical and oral bowel preparation, with 726 surgical site infections.
[b]The baseline value for wound classification is clean/contaminated procedures.
[c]The baseline value for ASA classification is an ASA score of 1 or 2.
[d]The baseline value for procedural group is colectomy procedures.
[e]The baseline value for surgical approach is open procedures.
[f]Surgical time divided by 15 minutes.

© 2026 Collins CD et al. *JAMA Network Open*.

eTable 6. Poisson Regression Model Results – Prophylaxis Regimens

Variable	Adjusted risk ratio (95% CI)	*P* value
**Prophylaxis received**[a]		
Cefoxitin	1.16 (1.00, 1.34)	.047
Ertapenem	1.20 (1.02, 1.40)	.03
Ceftriaxone and metronidazole	0.92 (0.68, 1.26)	.61
Ampicillin and sulbactam	1.02 (0.79, 1.33)	.88
Cefotetan	1.10 (0.84, 1.44)	.48
Metronidazole and fluoroquinolone	1.14 (0.88, 1.47)	.33
Clindamycin and aminoglycoside	1.64 (1.29, 2.08)	<.001
Metronidazole and aminoglycoside	1.61 (1.07, 2.41)	.02
Clindamycin and fluoroquinolone	2.06 (1.29, 3.30)	.002
Guideline-concordant dosing	1.03 (0.89, 1.19)	.68
Guideline-concordant timing	1.08 (0.88, 1.33)	.45
MOABP	0.61 (0.54, 0.68)	<.001
Age	0.99 (0.99, 1.00)	.001
Sex, female	0.98 (0.88, 1.09)	.73
Race, white	1.05 (0.92, 1.20)	.47
Body mass index	1.02 (1.02, 1.03)	<.001
Diabetes	1.05 (0.91, 1.21)	.49
Smoker	1.16 (1.02, 1.31)	.02
Corticosteroid use	1.27 (1.05, 1.54)	.01
**Wound classification**[b]		
Contaminated	1.31 (1.14, 1.51)	<.001
**ASA classification**[c]		
ASA 3 and 4	1.22 (1.08, 1.37)	.001
**Procedural group**[d]		
Other colon procedures	1.19 (1.04, 1.37)	.01
Proctectomy	1.40 (1.15, 1.71)	.001
**Surgical approach**[e]		
Laparoscopic	0.48 (0.41, 0.55)	<.001
Robotic	0.59 (0.50, 0.69)	<.001
Other	0.32 (0.12, 0.85)	.02
Surgery duration, minutes divided by 15[f]	1.04 (1.04, 1.05)	<.001

Abbreviations: CI, confidence interval; MOABP, mechanical and oral antibiotic bowel preparation; ASA, American Society of Anesthesiologists.
[a]The baseline value for prophylaxis received is cefazolin and metronidazole.
[b]The baseline value for wound classification is clean/contaminated procedures.
[c]The baseline value for ASA classification is an ASA score of 1 or 2.
[d]The baseline value for procedural group is colectomy procedures.
[e]The baseline value for surgical approach is open procedures.
[f]Surgical time divided by 15 minutes.

© 2026 Collins CD et al. *JAMA Network Open*.

eTable 7. Demographics and Clinical Characteristics in the Propensity Matched Cohort

Variable and Label[a]	β-lactam prophylaxis (n=2,042)	Alternative prophylaxis (n=2,042)	Standardized mean difference[f] (n=4,084)	*P* value
Age[b]	62.9 (13.7)	63.3 (13.5)	.03	.43
**Sex**			.11	.001
Female	1,212 (59.4)	1,317 (64.5)		
Male	830 (40.6)	725 (35.5)		
**Race[c]**			.06	.07
Other	394 (19.3)	349 (17.1)		
White	1,648 (80.7)	1,693 (82.9)		
Body mass index, kg/m^2 [b]	29.0 (6.5)	29.2 (6.8)	.03	.58
Diabetes	379 (18.6)	389 (19.0)	.01	.69
Smoker	388 (19.0)	397 (19.4)	.01	.72
Corticosteroid use	134 (6.6)	170 (8.3)	.07	.03
**Wound classification**			.03	.27
Clean/Contaminated	1,781 (87.2)	1,804 (88.3)		
Contaminated	261 (12.8)	238 (11.7)		
**ASA classification**			.06	.07
ASA 1 and 2	865 (42.4)	808 (39.6)		
ASA 3 and 4	1,177 (57.6)	1,234 (60.4)		
**Procedural group**				.76
Colectomy	1,599 (78.3)	1,585 (77.6)	.02	
Other colon procedures	314 (15.4)	331 (16.2)	.02	
Proctectomy	129 (6.3)	126 (6.2)	.01	
**Surgical approach**				.14
Open	887 (43.4)	936 (45.8)	.05	
Laparoscopic	786 (38.5)	714 (35.0)	.07	
Robotic	356 (17.4)	378 (18.5)	.03	
Other	13 (0.6)	14 (0.7)	.01	
MOABP	1,449 (71.0)	1,394 (68.3)	.06	.06
Surgery duration, minutes divided by 15[d, e]	10 (7, 14)	10 (7, 14)	.02	.31
**Prophylaxis received**				<.001
Cefazolin and metronidazole	958 (46.9)			
Cefoxitin	421 (20.6)			
Ertapenem	347 (17.0)			
Ceftriaxone and metronidazole	102 (5.0)			
Ampicillin and sulbactam	99 (4.8)			
Cefotetan	115 (5.6)			
Metronidazole and fluoroquinolone		896 (43.9)		
Clindamycin and aminoglycoside		787 (38.5)		
Metronidazole and aminoglycoside		237 (11.6)		
Clindamycin and fluoroquinolone		122 (6.0)		
**Guideline-concordant dosing and timing**				
Guideline-concordant dosing	1,690 (82.8)	1,084 (53.1)	.67	<.001
Guideline-concordant timing	1,937 (94.9)	1,707 (83.6)	.37	<.001
Guideline-concordant dosing and timing	1,610 (78.8)	751 (36.8)	.94	<.001
At least one preoperative antibiotic post-incision	54 (2.6)	198 (9.7)	.3	<.001

© 2026 Collins CD et al. *JAMA Network Open*.

All preoperative prophylaxis post-incision	20 (1.0)	11 (0.5)	.05	.11

Abbreviations: ASA, American Society of Anesthesiologists; MOABP, mechanical and oral antibiotic bowel preparation; SD, standard deviation.
[a]Data are No. (%) unless otherwise specified.
[b]Data are summarized as means (SD).
[c]Race was recorded in the MSQC registry. “Other” includes American Indian or Alaska Native, Asian, Black or African American, Native Hawaiian or Other Pacific Islander, and Unknown race.
[d]Data are summarized as median (interquartile range).
[e]Surgical time divided by and rounded to the nearest 15 minutes.
[f]Data are presented as absolute standardized mean difference (SMD). For continuous variables, SMDs were calculated using group means and SDs, even when medians (interquartile ranges) are displayed for descriptive purposes.

© 2026 Collins CD et al. *JAMA Network Open*.

eTable 8. Outcomes by Cohort in the Propensity Matched Cohort

Variable[a]	**β-lactam prophylaxis (n=2,042)**	**Alternative prophylaxis (n=2,042)**	**Standardized mean difference[b] (n=4,084)**	***P* value**
**Surgical site infections**				
Overall study population	123 (6.0)	172 (8.4)	.09	.003
Deep surgical site infection	26 (1.3)	26 (1.3)	0	>.99
Organ space surgical site infection	45 (2.2)	66 (3.2)	.06	.04
Superficial surgical site infection	53 (2.6)	83 (4.1)	.08	.009
Clostridioides difficile infection	13 (0.6)	24 (1.2)	.06	.07

Abbreviations: MOABP, mechanical and oral antibiotic bowel preparation.
[a]Data are No. (%).
[b]Data are presented as absolute standardized mean difference (SMD).

© 2026 Collins CD et al. *JAMA Network Open*.

eTable 9. Association with Surgical Site Infection (Modified Poisson Regression) in the Propensity Matched Cohort

**Variable**	**Adjusted risk ratio (95% CI)**	***P* value**
β-lactam prophylaxis	0.74 (0.59, 0.94)	.01
Guideline-concordant dosing	1.09 (0.86, 1.38)	.48
Guideline-concordant timing	1.10 (0.79, 1.53)	.59
MOABP	0.65 (0.52, 0.82)	<.001
Age	1.00 (0.99, 1.01)	.37
Sex, female	1.17 (0.93, 1.47)	.18
Race, white	1.15 (0.86, 1.54)	.35
Body mass index	1.02 (1.01, 1.04)	.005
Diabetes	1.42 (1.10, 1.84)	.008
Smoker	1.31 (1.01, 1.69)	.04
Corticosteroid use	1.16 (0.82, 1.66)	.39
**Wound classification**[a]		
Contaminated	1.40 (1.05, 1.85)	.02
**ASA classification**[b]		
ASA 3 and 4	1.09 (0.85, 1.41)	.49
**Procedural group**[c]		
Other colon procedures	1.33 (1.01, 1.75)	.04
Proctectomy	1.05 (0.65, 1.69)	.84
**Surgical approach**[d]		
Laparoscopic	0.42 (0.31, 0.57)	<.001
Robotic	0.44 (0.31, 0.63)	<.001
Other	0.98 (0.24, 4.02)	.97
Surgery duration, minutes divided by 15[e]	1.06 (1.04, 1.08)	<.001

Abbreviations: CI, confidence interval; MOABP, mechanical and oral antibiotic bowel preparation; ASA, American Society of Anesthesiologists.

[a]The baseline value for wound classification is clean/contaminated procedures.
[b]The baseline value for ASA classification is an ASA score of 1 or 2.
[c]The baseline value for procedural group is colectomy procedures.
[d]The baseline value for surgical approach is open procedures.
[e]Surgical time divided by 15 minutes.

© 2026 Collins CD et al. *JAMA Network Open*.
