# Supplementary material for: β-Lactam vs Non–β-Lactam Prophylaxis in Elective Colorectal Surgery
Source: JAMA Netw Open. 2026 Apr 13;9(4):e266708. doi: 10.1001/jamanetworkopen.2026.6708 (PMC13077511; doi:10.1001/jamanetworkopen.2026.6708)
Supplement: Supplement 2. — Data Sharing Statement [file jamanetwopen-e266708-s002.pdf]

Data Sharing Statement

Collins. β-Lactam vs Non–β-Lactam Prophylaxis in Elective Colorectal Surgery. *JAMA Netw Open*. Published April 13, 2026. doi:10.1001/jamanetworkopen.2026.6708

Data
Data available: No

Additional Information
Explanation for why data not available: Data was obtained through the Michigan Surgical Quality Collaborative.
